# Metal Halide Solid-State Surface Treatment for High Efficiency PbS and PbSe QD Solar Cells

**DOI:** 10.1038/srep09945

**Published:** 2015-04-24

**Authors:** Ryan W. Crisp, Daniel M. Kroupa, Ashley R. Marshall, Elisa M. Miller, Jianbing Zhang, Matthew C. Beard, Joseph M. Luther

**Affiliations:** 1 National Renewable Energy Laboratory, Golden, CO 80401 USA; 2 Department of Physics, Colorado School of Mines, Golden, CO 80401 USA; 3 Department of Chemistry and Biochemistry, University of Colorado, Boulder, CO 80309 USA; 4 School of Optical and Electronic Information, Huazhong University of Science and Technology, Hubei 430074, China

## Abstract

We developed a layer-by-layer method of preparing PbE (E = S or Se) quantum dot (QD) solar cells using metal halide (PbI_2_, PbCl_2_, CdI_2_, or CdCl_2_) salts dissolved in dimethylformamide to displace oleate surface ligands and form conductive QD solids. The resulting QD solids have a significant reduction in the carbon content compared to films treated with thiols and organic halides. We find that the PbI_2_ treatment is the most successful in removing alkyl surface ligands and also replaces most surface bound Cl^-^ with I^-^. The treatment protocol results in PbS QD films exhibiting a deeper work function and band positions than other ligand exchanges reported previously. The method developed here produces solar cells that perform well even at film thicknesses approaching a micron, indicating improved carrier transport in the QD films. We demonstrate QD solar cells based on PbI_2_ with power conversion efficiencies above 7%.

Solution-processed photovoltaics (PV) represent a promising route forward in reducing the cost of solar energy production. Quantum dot (QD) solids are one such solution-processed system currently being researched. In addition to being solution processable, QD solar cells (QDSCs) have a higher limiting single junction power conversion efficiency than is possible using conventional bulk or thin film semiconductors due to enhanced multiple exciton generation (MEG) in the QDs^1^,^2^. Recent improvements in QDSC performance and processing ease have resulted from modification of the device architecture, processing of the QD-layers under ambient conditions, improved QD synthetic procedures and surface treatments improving QD passivation^3^,^4^,^5^,^6^. A critical component of the progress listed above is the incorporation of halides into the QD matrix^6^,^7^. Specifically, Cl^-^ anions were shown to improve stability while passivating trap states that lower the minority-carrier lifetime^3^,^4^,^8^. Incorporation of these halide anions has been achieved by using chloride precursors in the QD synthesis^3^, using post-synthesis solution treatments^9^,^10^,^11^, and, recently, employing ammonium halide salts as the only ligand treatment^6^,^12^. However, when using the previously reported halide passivation schemes, organic molecules that are instrumental in delivering the halide anion (e.g. tetrabutylammonium iodide (TBAI), 3-chloropropane-1-thiol, methylammonium iodide (MAI), *etc.*), leave behind cationic organic residue that could potentially limit device performance. In contrast, here, we demonstrate a procedure that removes nearly all of the organic moieties from the QD solid during device fabrication. The groups of Wang, Talapin, and Kovalenko have previously reported solution-phase ligand exchanges using halide ligands but have not applied them to solar cells^13^,^14^,^15^. Our procedure is based upon a layer-by-layer approach demonstrated previously but uses metal halides dissolved in dimethlyformamide (DMF) (rather than thiols in acetonitrile or alcohols) to build-up thick, all-inorganic films by either dip coating or spin coating with PbS or PbSe QDs.

## Results

The QD synthesis used in this study follows previous reports where PbE (E = S, Se) QDs are made by cation exchange of CdE QDs with PbCl_2_/oleylamine^4^. Of the numerous metal halide materials available, we focus on four metal halides solvated in DMF: PbCl_2_, PbI_2_, CdCl_2_, and CdI_2_. The chosen metal halides introduce ions (Cl^-^ or I^-^) that have demonstrated passivation of QD trap states. Metal halides have not previously been used as the sole ligand treatment for QDSCs but rather as a pre- or post-treatment in conjunction with short-chained alkyl thiol ligands. Previous reports indicate soaking QD solids in neat DMF displaces the native oleate ligands derived from oleic acid (OA) and leads to oriented attachment along the (100) facets of the PbE QDs^16^. Here, we find that the metal-halide:DMF treatment removes Pb-oleate from the QDs while incorporating the metal halide into the film as is discussed below.

**Figure 1:**
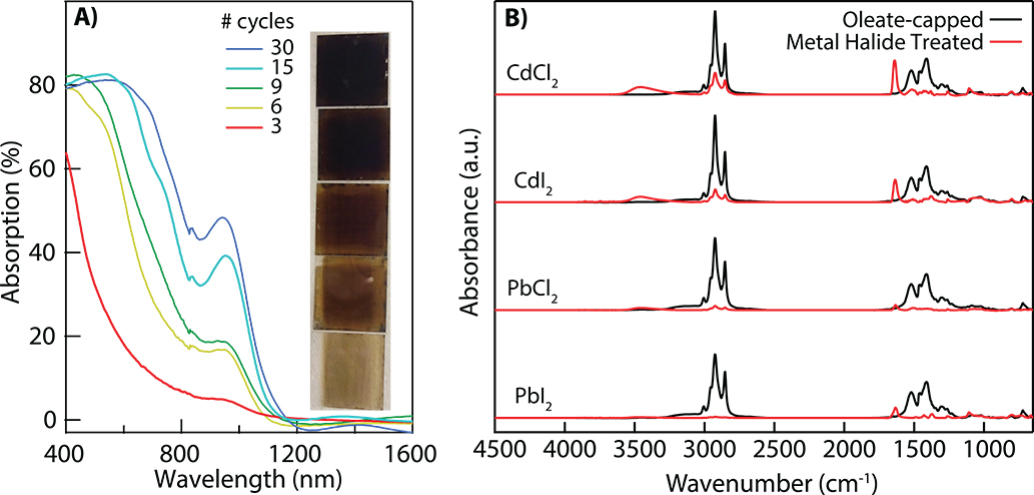
A) Absorption spectra calculated by measuring the transmission and reflection spectra of dip coated PbS QD films on glass as a function of the number of dip cycles using PbI_2_. Inset: photograph of PbS QD films with variable thickness controlled by the number of dip cycles given in the legend. B) Fourier-Transform Infrared (FTIR) spectra of 1.3 eV bandgap PbS QDs dropcast from hexane (black traces) and then soaked for 2 hours in 10 mM metal halide in *N,N*-dimethylformamide (DMF) (red traces). The peak at 1640 cm^−1^ is attributed to residual DMF that can be removed with heating and/or placing the film under vacuum.

Dip coating QDs allows for a controlled thickness of a compact film with appropriate surface coverage^17^. In Fig. 1A, we show the increase in absorption of PbS QD films with increasing number of deposition cycles while preserving the first exciton feature originating from the individual QD size. In Fig. 1B, we show baseline-corrected Fourier-Transform Infrared (FTIR) spectra of dropcast films capped with the native oleate ligand (black lines) and the corresponding spectra after ligand treatment with various metal halide salts in DMF (red lines). Based on the ratios of the largest absorbance feature at 2925 cm^−1 ^(corresponding to the *ν_a_* (–CH_2_) mode), the iodide salts remove more Pb-oleate than their respective chloride salts (i.e. CdI_2_ removes more than CdCl_2_), and the lead salts remove more than the cadmium salts (i.e PbI_2_ is more effective than CdI_2_). This trend is deduced using a ratio of the absorbance at 2925 cm^−1^ i.e. [post-soak]/[pre-metal halide soak]; 26% oleate remains after treatment with CdCl_2_, 14% after CdI_2_ treatment, 5.1% after PbCl_2_ treatment, and 1.4% after PbI_2_ treatment. However, some of the residual organics from DMF are still present after rinsing, as indicated by the peak near 1640 cm^−1^. The generality of the concept is shown by using each of the compounds in Fig. 1B, but focusing on PbI_2_ in DMF as a treatment to prepare QD solar cells since it is most effective at removing the oleate.

To further detail the composition and properties of the QD films treated with PbI_2_ (PbS_PbI2_), we examined the atomic concentrations and energy levels using x-ray photoelectron spectroscopy (XPS) and compare to that of other ligand-exchanged QD films. We fabricate films using iodine-containing ligands: TBAI and PbI_2_, as well as the sulfur-containing ligands: MPA and NH_4_SCN. Both NH_4_SCN and MPA have carbon signatures greater than 20%. Comparing QD films treated with TBAI to those treated with PbI_2_ in Table 1, we find that the percentage of carbon present in the film is greatly reduced (from 26.7% to 2.5%) when using the PbI_2_ treatment. Interestingly, the MPA and NH_4_SCN do not displace the Cl present in the QDs (Cl added during the ion exchange reaction *via* PbCl_2_/oleylamine^4^), whereas after treating QD films with TBAI or PbI_2_, Cl is not detected by XPS. This demonstrates the strong bonding character of iodine to the surface of lead chalcogenide quantum dots and may be responsible for the lessened sensitivity to oxygen in PbS and PbSe devices found here and previously^6^.

**Figure 2:**
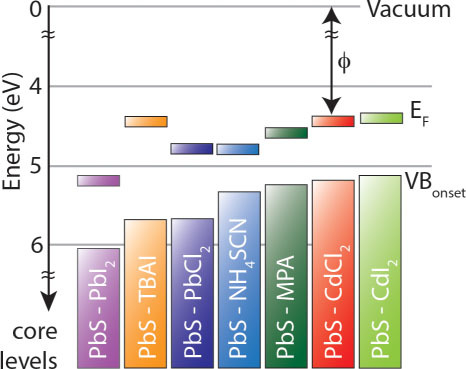
Summary of photoelectron spectroscopy results of 1.3 eV bandgap PbS QDs with various surface treatments. The E_F _- E_VB onset_ and ϕ of PbS QDs changes with PbI_2_, TBAI, PbCl_2_, NH_4_SCN, MPA, CdCl_2_, and CdI_2_ surface treatments/ligand exchange.

Researchers have demonstrated a link between the stoichiometry in ionic QDs and majority carrier type in QD films^18^,^19^. For instance, the Pb:E ratio decreased with the addition of chalcogens from ligands like MPA or NH_4_SCN resulting in p-type QD films^18^,^19^,^20^. Changes in the stoichiometry would therefore alter the Fermi level position within the bandgap. The XPS spectra can be used to determine the work function (ϕ = difference between Fermi energy and vacuum level) and the onset of emitted electrons from VB states relative to the Fermi energy (E_F_ – E_VB onset_). Here we find that treating the QDs with MPA or NH_4_SCN decreases the Pb:S ratio compared to the I^-^ treated films and the separation between the onset of the valence band (VB) states and the Fermi level is also smaller, consistent with previous reports^21^. In addition, the I^-^ treatments lead to a deeper VB (i.e. larger energy difference between vacuum and the VB onset) than the sulfur-containing ligand treatments as shown in Fig. 2. With total cation:anion ratio equal to unity, the lower Pb:S ratios for the MPA and NH_4_SCN treatments compared to the I^-^ ligand treatment support the conclusion that the MPA and NH_4_SCN treatments lead to more *p*-type films than the I^-^ ligand treatments^22^.

The XPS results of the VB onset and ϕ show that the ligand can dictate the Fermi level position within the bandgap *and* can control the overall band positions relative to vacuum^21^,^23^,^24^. Our results with the TBAI, MPA, and NH_4_SCN ligand treatments agree with those of Brown et al.^21^, and we find (Fig. 2) that the PbI_2_ ligand exchanged PbS QD film has the lowest lying VB onset and ϕ of all of the ligands studied here and in previous work with PbS QD films. Furthermore, treating the PbS QDs with CdCl_2_ or CdI_2_ yields a shallower valence band onset. Control over both the band positions and majority carrier type within QD solids enables deliberate engineering of the energetics within a device.

**Figure 3:**
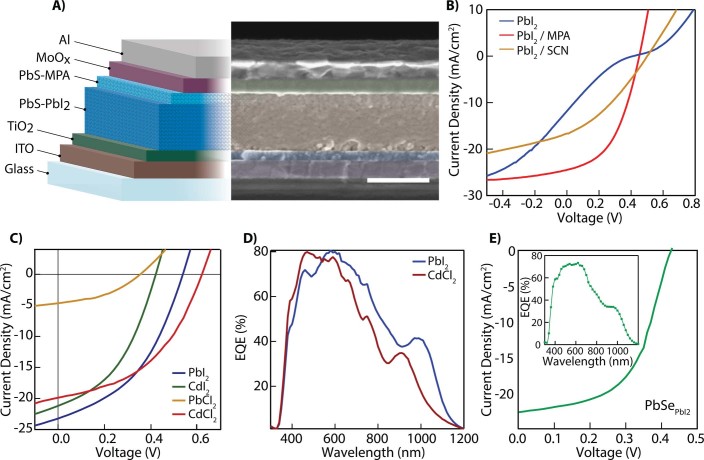
A) Schematic representation of the device structure superimposed on a false-color scanning electron microscope image for a completed PbS_PbI2_/PbS_MPA_ device. Scale bar is 500 nm. B) Current-voltage characteristics using only a PbI_2_ treatment shows low FF (blue trace) but using a secondary layer treated with MPA (red trace) and with the inorganic SCN^-^ ligand (gold trace) aids in band alignment yielding improved FF and PCE. C) Current-voltage characteristics of devices incorporating the four metal halides discussed above are shown. Using CdCl_2_ as opposed to PbI_2_ improves the V_OC_ to over 615 mV. D) External quantum efficiency (EQE) curves for PbS QDSCs with PbI_2_ and CdCl_2_ ligand treatments (PbS_MPA_ is the back layer as shown in panel A). E) Current-voltage characteristics of air-fabricated PbSe_PbI2_ QDSC. The inset shows the external quantum efficiency for the device.

We then fabricated PbE QDSCs with the structure shown in Fig. 3A. The processing details and characteristics of devices made for this study are summarized in Table 2. The devices are fabricated in air using a layer-by-layer coating process. Both dip coating and spin coating yield nominally the same results with details given in Table 2 and the Methods section. Both of these deposition protocols allow for conformal films with well-controlled thickness. As mentioned above, a unique feature of QD solids is the ability to control the absolute energy levels by applying different ligands. This effect has been attributed to ligand-induced surface dipoles^21^. Such control allows the energetics within a device to be engineered by using multiple surface treatments during the QD deposition to create bilayer (or in principle, more complex) structures of QD solids. For example, Semonin *et al.* demonstrated increased performance in PbSe solar cells by stacking ethanedithiol- (EDT) and hydrazine-treated layers^1^. Other combinations using TBAI and EDT or tetramethylammoinium hydroxide have been used to enhance carrier collection resulting in improved device performance^6^,^25^. In Fig. 3B, we compare devices with only a PbS_PbI2_ layer to those with bilayer structures where the PbS_PbI2_ layer is followed by either PbS_MPA_ or PbS_NH4SCN_ layers and find that the bilayer structure can greatly improve the current density-voltage (*J*-*V*) characteristics of the device. Although PbS QDs treated with the inorganic SCN^-^ ligand have been reported to be more conductive in QD films than the organic MPA ligand^26^,^27^, we find that the QD devices presented here function more efficiently with PbS_PbI2_/PbS_MPA_ than PbS_PbI2_/PbS_NH4SCN_.

Adopting the PbS_PbI2_/PbS_MPA_ bilayer structure, we then compare PV devices using each of the metal halides discussed. Figure 3A shows a scanning electron microscopy (SEM) image of a completed PbS_PbI2_/PbS_MPA_ device indicating highly uniform QD deposition throughout the device. The difference in contrast shown in the SEM for the PbI_2_- *vs.* MPA-capped QDs indicates that the layers remain distinct with likely different material density or perhaps conductivity. While we have optimized the device fabrication conditions for the PbI_2_ treatment, we note that each of the metal halides results in functioning devices and each affect the PV performance in unique ways. For example, in Fig. 3C, we show that devices fabricated using CdCl_2_ have an improved open circuit voltage over those fabricated from PbI_2_-treated QDs and reach a PCE of 5.6%. The spectral response of a CdCl_2_-treated device (Fig. 3D) exhibits a 100-nm blue shift in the wavelength of the first exciton feature that is likely due to a surface ion exchange which reduces the size of the PbS core and increases the bandgap^28^. Metal halide treatments can also be used to fabricate PbSe QDSCs under ambient conditions (PbSe is generally more prone to oxidation than PbS). The *J*-*V* characteristics of a 1.3 eV bandgap PbSe QDs device are shown in Fig. 3E with the inset showing the external quantum efficiency (EQE) of the device with >70% response throughout most of the visible spectrum.

**Figure 4:**
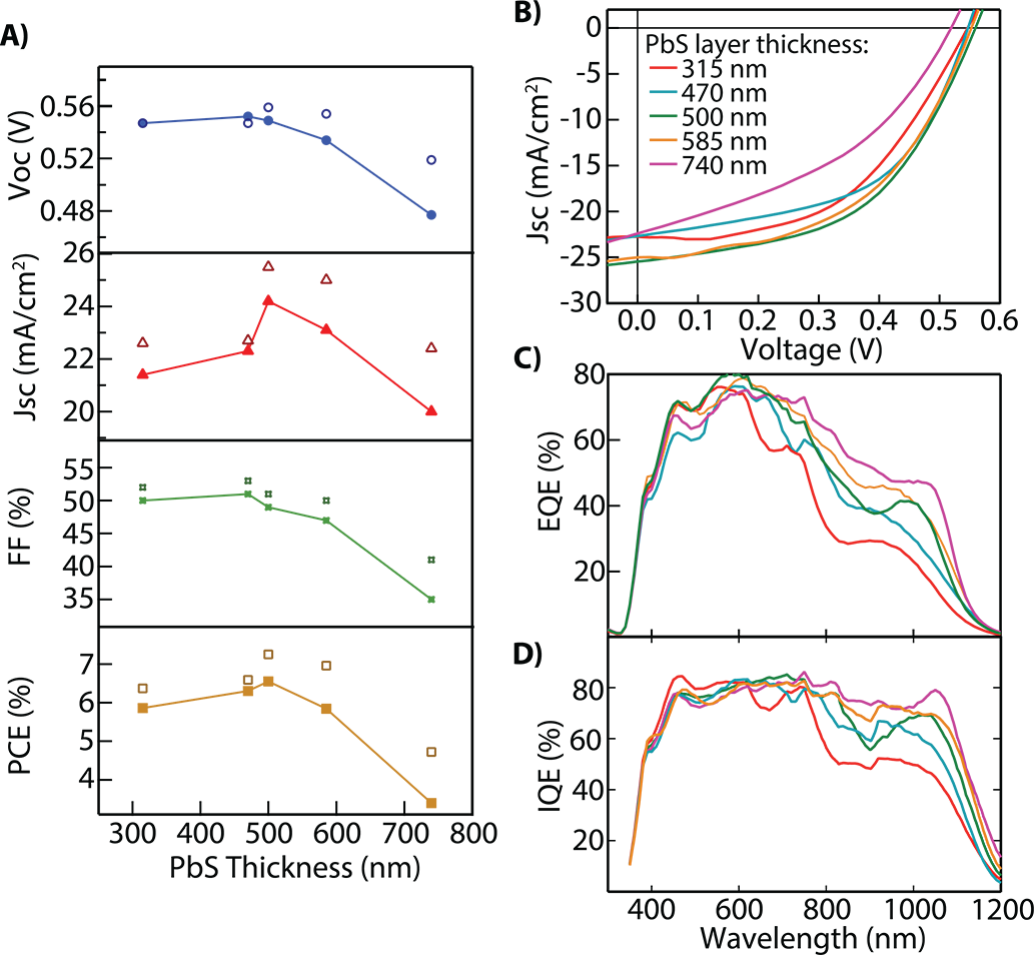
A) V_OC_, J_SC_, FF, and PCE plotted as a function of device thickness for spincoated PbI_2_-treated devices. Solid symbols represent the average of 6 devices and hollow symbols are the best devices. B) *J-V* curves of cells shown in panel A with the best cell reaching a PCE of 7.25% with 500 nm thickness of the QD layer. C) EQE response of cells showing improved response of longer wavelength light as the cell absorbs more light. D) Internal quantum efficiency (IQE) determined by dividing the EQE by the absorption. The color coding is consistent in panels B-D and annotated in the legend of panel B.

We test the thickness dependence of the PbS absorber layer by producing devices composed of 4, 6, 7, 8, and 10 sequential spin coating steps. After each spin, the film is treated by soaking in 10 mM PbI_2_ in DMF for 3 minutes. The last two coatings were treated by 10% MPA in methanol rather than PbI_2_. In Fig. 4A, we plot the open-circuit voltage (V_OC_), short-circuit current (J_SC_), fill factor (FF), and PCE as a function of the total QD layer thickness. The solid symbols represent the average of 6 devices and the hollow symbols represent the champion device for each film thickness. Current-voltage characteristics of the devices are shown in Fig. 4B with the best device reaching a PCE of 7.25% which corresponds to a thickness of 500 nm. Figure 4C shows the EQE response of the devices as a function of thickness and indicates a general trend of increasing spectral response for lower energy photons (*i.e.* photons with wavelength between 600 and 1200 nm). We also determined that the internal quantum efficiency (IQE) increases in this same manner with the thickest cell showing a flat response of 80–85% (Fig. 4D). Ideally, the IQE should be independent of the cell thickness unless there is high carrier recombination at the back interface. For the device with a PbS QD thickness of 740 nm, the IQE is roughly 80% for all photons absorbed in the QD layer (*i.e.* photon energy above the bandgap of the PbS QDs and below the absorption of the glass/ITO substrate). Electron transport is sufficient to extract 80% of carriers generated in the device, indicated by the IQE and flat spectrum, despite being significantly thicker than the highest efficiency reported PbS QDSC^6^. The PbI_2_ treatment described here is, therefore, very promising for improving the overall efficiency of QDSCs as the J_SC_ only begins to drop as the film thickness approaches 740 nm.

To conclude, we present metal halide treated films of PbE yielding high efficiency devices. This inorganic ligand treatment allows for relatively thick films (~600 nm) to be incorporated into devices while still maintaining good transport (i.e. high current) in the device. The XPS results highlight the control over the PbS QD absorber layer by choice of ligand. We have shown with XPS that different chemical treatments affect the QD surface, and subsequently, how these surface treatments directly control the energy levels of the QD absorber layer. Additionally, XPS and FTIR analysis confirmed that the metal halide exchange lessens the residual organic elements in the film. Furthermore, using PbS QDs as the low bandgap cell in tandem configurations where better collection of the near infrared photons is needed is now more feasible as collection efficiency throughout nearly the entire spectrum exceeds 50% with an absorber thickness >700 nm.

## Methods

The QDs were synthesized following a previously published procedure^4^. For PbSe, CdSe was first synthesized following a modified version of the procedure published by Pu *et al*.^29^ to obtain ~5 nm, monodisperse CdSe. The CdSe was then chemically converted to PbSe through a cation exchange reaction by mixing 0.834 g PbCl_2_ with 10 mL oleylamine (OLA), degassing, and heating to 140°C for 30 min. The mixture was then heated to 190°C and 2 mL of CdSe (100 mg/mL, in ODE) is injected. The reaction was left at 180°C for 30 seconds then quenched with a water bath. As the reaction cools, 10 mL hexane and 8 mL OA are injected at 70°C and 30°C, respectively. The reaction was allowed to cool and the QDs were washed by precipitation-redispersion with ethanol and hexane. The final dispersion was centrifuged to remove any excess chloride salts and filtered through a 0.2 μm Nylon filter.

PbS was synthesized by the cation exchange of CdS. CdS was synthesized following the procedure published by Zhang et al.^30^. The cation exchange follows that of the CdSe, except the precursors are cooled to 90°C before the injection of CdS (150 mg/mL in toluene) and the reaction runs for 60 seconds. The product was washed and filtered in the same way as described above.

The FTIR absorbance measurements were taken on a Thermo-Nicolet 6700 FT-IR spectrometer in transmission mode with a resolution of 4 cm^−1^. Clean Si plates were used for background measurements, and films of OA-capped QDs were drop cast onto the Si plates for the oleate-capped measurements. The samples were then submerged in 10 mM metal halide in DMF solutions for 2 hours and rinsed with acetonitrile. These metal halide treated samples were then measured, and spectra with sloping baselines were baseline-corrected.

The XPS measurements were performed on a Physical Electronics, Inc. 5600 ESCA instrument, which has been discussed in detail previously^31^. Briefly, the radiation is produced by a monochromatic Al (Kα) source centered at 1486.6 eV. The VB spectra were taken with a step size of 0.05 eV and a pass energy of 5.85 eV. The electron binding energy scale was calibrated using the Fermi edge of cleaned metallic substrates (Au, Mo, Cu, and/or Ag), giving the spectra an uncertainty of ±0.05 eV. We verify that charging during the photoemission experiments is insignificant by measuring the X-ray power dependence of various spectral features (core levels, VBMs, and/or secondary electron cutoffs). We find the VB onset by determining the intersection between the baseline and a linear fit to the main VB feature^32^.

Solar cell fabrication consisted of dip coating or spin-coating on patterned ITO-coated glass slides from Thin Film Devices where we first deposited a TiO_2_ layer with a sol-gel method. TiO_2_ sol-gel was prepared in air by mixing 5 mL anhydrous ethanol, 2 drops hydrochloric acid, and 125 μL DI water. This mixture was stirred while 375 μL titanium ethoxide is added drop-wise to ensure that no precipitates form. This yielded a clear liquid that was stirred for 48 hours with the headspace of the vial filled with nitrogen. It was then stored in a freezer until needed. The ITO/glass substrates were cleaned vigorously with ethanol and UV-ozone treated before depositing TiO_2_. Within 10 min of UV-ozone treatment, 70 μL TiO_2_ sol-gel was spun at 1400 RPM for 30 sec. The TiO_2_ is wiped off the ITO contact pads using ethanol and the films are dried at 115°C then annealed at 450°C for 30 min. The films are stored in air and sit in air for at least 1 day before use. For dip coating, immersing the substrates into a ~15 mg/mL solution of QDs in hexane and smoothly removing them leaves a thin film of QDs as discussed previously^17^. Dipping this film into the 10 mM metal halide/DMF solution for 30–60 seconds renders the QD layer insoluble in hexane and allows for thick films to be built up layer-by-layer (where the term “layer” does not imply a monolayer of QDs, but rather one coating of QDs). A post-ligand treatment with neat acetonitrile (ACN) was necessary to remove the DMF because the residual DMF does not dry rapidly. It should be noted that the metal halides discussed here are not appreciably soluble in ACN making ACN a poor choice of solvent for the ligand exchange. A mixture of 20 vol.% DMF/ACN solvated the PbI_2_ and devices made in this fashion performed nearly as well as those with PbI_2_ in DMF for the ligand treatment solvent (Table 2). Typical dip coated devices used 10–15 layers of PbI_2_-treated QDs followed by 3–4 layers treated with 10% MPA in methanol (MeOH) or alternatively a 10 mM solution in MeOH was used for the NH_4_SCN treatment. Spin coated devices used a variable number of layers for the PbI_2_-treatment as discussed in the manuscript with 2 layers of MPA-capped QDs in each case. The QDs were dispersed in octane at a concentration of 40 mg/mL and spun at 1000 rpm for 45 s before being immersed in 10 mM metal halide solution for 3 minutes and rinsed with ACN. The last 2 cycles of QDs were treated with 10% MPA in MeOH by dipping the device into a MPA/MeOH solution, rinsing twice with MeOH and drying with nitrogen. All devices presented here were fabricated at room temperature (~23.9–26.7°C) and relative humidity that fluctuates between 16–20%. A MoO_x_/Al back contact was then thermally evaporated as discussed by Gao *et al.*^33^.

Device testing was carried out using Newport solar simulators adjusted by measuring a calibrated Si photodiode reference to match the AM1.5 spectrum. Some devices were tested in glovebox atmosphere while others were tested in air; details annotated in Table 2. Device area is 0.11 cm^2^ but an aperture of 0.059 cm^2^ was used to define the active illuminated area. Spectral response measurements were performed on an Oriel IQE-200 system.

## Additional information

**Competing financial interests:** The authors declare no competing financial interests.

## Figures and Tables

**Table 1:** Relative atomic percentage of elements in ligand-exchanged QD films determined by XPS

Treatment	C	N	I	Cl	Pb	S	O	Cd	Pb:S	Pb:I	Pb:Cl	(Pb+Cd):(S+I+Cl)
PbI_2_	2.5	*	26.9	*	45.7	19.3	5.3	0.3	2.1	2.1		1.0
TBAI	26.7	1.3	19.2	*	34.3	16.4	1.7	0.4	2.1	1.8		1.0
MPA	27.3	*	*	7.0	28.4	19.7	16.6	1.0	1.4		4.1	1.1
NH_4_SCN	21.0	2.9	*	4.0	37.8	27.3	7.0	0.2	1.4		9.5	1.2

*values below detection limit

**Table 2:** Compilation of the various device parameters explored.

QDs (precursor)	n-type contact	Ligand/solvent	Back surface ligand	Deposition method	PbE thickness	Voc (mV)	Jsc (mA/cm^2^)	FF (%)	PCE (%)	Measurement environment
PbSe (CdSe)	TiO_2_	PbI_2_/DMF	MPA (10%)	Dipcoat	300 nm	428	22.7	54.8	5.3	N_2_
PbS (CdS)	CdS	PbI_2_/DMF	MPA (10%)	Dipcoat	350 nm	543	16.5	45.0	4.0	Air
PbS (CdS)	TiO_2_	PbI_2_/DMF:ACN 1:5	EDT (1 mM)	Dipcoat	300 nm	623	14.2	36.2	3.2	Air
PbS (CdS)	In:ZnO sol-gel	PbI_2_/DMF:ACN 1:5	EDT (1 mM)	Dipcoat	550 nm	567	17.4	42.8	4.2	Air
PbS (CdS)	ZnO NCs	PbI_2_/DMF:ACN 1:5	EDT (1 mM)	Spincoat	500 nm	606	20.5	34.0	4.2	Air
PbS (CdS)	TiO_2_	PbI_2_/DMF:ACN 1:5	MPA (10%)	Dipcoat	550 nm	597	21.8	45.0	5.9	Air
PbS (CdS)	TiO_2_	MPA	N/A	Dipcoat	400 nm	542	6.96	47.1	1.8	Air
PbS (CdS)	TiO_2_	EDT	N/A	Dipcoat	550 nm	596	15.8	33.8	3.2	Air
PbS (CdS)	TiO_2_	PbI_2_/DMF	MPA (10%)	Dipcoat	350 nm	584	25.3	44.8	6.6	Air
PbS (CdS)	TiO_2_	PbI_2_/DMF	MPA (2%)	Spincoat	420 nm	496	23.0	43.2	4.9	N_2_
PbS (CdS)	TiO_2_	PbI_2_/DMF:ACN 1:5	Na_2_S (10 mM)	Dipcoat	400 nm	513	11.9	19.9	1.2	N_2_
PbS (CdS)	TiO_2_	PbI_2_/DMF:ACN 1:5	NH_4_SCN (10 mM)	Dipcoat	340 nm	500	16.6	33.2	2.8	N_2_
PbS (CdS)	TiO_2_	PbI_2_/DMF:ACN 1:5	MPA (10%)	Dipcoat	750 nm	516	14.1	48.6	3.5	N_2_
PbS (PbCl_2_)	TiO_2_	PbI_2_/DMF	MPA (10%)	Spincoat	550 nm	476	22.8	42.3	4.6	N_2_
PbS (PbCl_2_)	TiO_2_	PbI_2_/DMF	MPA (10%)	Spincoat	300 nm	466	21.2	40.4	4.0	N_2_
PbS (PbCl_2_)	TiO_2_	PbI_2_/DMF	MPA (10%)	Spincoat	300 nm	348	18.8	30.6	2.0	N_2_
PbS (CdS)	TiO_2_	PbI_2_/DMF	N/A	Spincoat	500 nm	412	11.8	18.9	0.9	N_2_
PbS (CdS)	TiO_2_	PbI_2_/DMF	MPA (10%) - dip	Spincoat	500 nm	431	19.5	30.5	2.6	N_2_
PbS (CdS)	TiO_2_	PbI_2_/DMF	MPA (10%)	Spincoat	500 nm	438	23.9	47.5	5.0	N_2_
PbS (CdS)	TiO_2_	PbI_2_/DMF	MPA (10%)	Spincoat	315 nm	547	22.6	52.0	6.4	N_2_
PbS (CdS)	TiO_2_	PbI_2_/DMF	MPA (10%)	Spincoat	470 nm	547	22.7	53.0	6.6	N_2_
PbS (CdS)	TiO_2_	PbI_2_/DMF	MPA (10%)	Spincoat	500 nm	559	25.5	51.0	7.3	N_2_
PbS (CdS)	TiO_2_	PbI_2_/DMF	MPA (10%)	Spincoat	585 nm	554	25.0	50.0	7.0	N_2_
PbS (CdS)	TiO_2_	PbI_2_/DMF	MPA (10%)	Spincoat	740 nm	519	22.4	41.0	4.7	N_2_
PbS (CdS)	TiO_2_	PbCl_2_/DMF	MPA (10%)	Spincoat	580 nm	354	4.66	41.0	0.7	N_2_
PbS (CdS)	TiO_2_	CdI_2_/DMF	MPA (10%)	Spincoat	580 nm	421	21.2	42.1	3.8	N_2_
PbS (CdS)	TiO_2_	CdCl_2_/DMF	MPA (10%)	Spincoat	580 nm	620	19.9	45.1	5.6	N_2_
